# Biomechanical Analysis of the Fixation Strength of a Novel Plate for Greater Tuberosity Fractures

**DOI:** 10.2174/1874325001812010218

**Published:** 2018-06-29

**Authors:** Aristotelis Kaisidis, Panagiotis G. Pantos, Dimitrios Bochlos, Horst Lindner

**Affiliations:** 1 Shoulder Department, Klinik Maingau, Rotes Kreuz,Frankfurt am Main,Germany; 2 Königsee Implantate GmbH, Aschau,Germany

**Keywords:** Biomechanical analysis, Greater tuberosity fractures, Kaisidis plate, Supraspinatus tendon, Solidworks 2015, Finite Element Method

## Abstract

**Background:**

The incidence of isolated greater tuberosity fractures has been estimated to be 20% of all proximal humeral fractures. It is generally accepted that displaced (>5 mm) fractures should be treated surgically but the optimal surgical fixation of greater tuberosity fractures remains unclear.

**Objective:**

The goal of this study was to simulate the environment of application of a new plate system (Kaisidis plate, Fa Königsee) for fractures of greater tuberosity, and to demonstrate the stability of the plate.

**Methods:**

A Finite Element Method (FEM) simulation analysis was performed on a Kaisidis plate fixed with nine screws, in a greater tuberosity fracture model. Solid Works 2015 simulation software was used for the analysis. The Kaisidis plate is a bone plate intended for greater tuberosity fractures. It is a low profile plate with nine holes for 2,4 mm diameter locking screws, eight suture holes and additional K-wire holes for temporary fixation of the fragment.

The supraspinatus tendon has the greatest effect on the fracture zone, and as such, was the primary focus for this study. For this study, we performed only linear calculations.

**Results:**

The calculations were performed in a way so that the total applied force resulted in a maximum stress of 816 N/mm^2^. The findings indicated that the most critical points of the Kaisidis system are the screws that are connected to the bone. The maximal force generated by the supraspinatus tendon was 784 N, which is higher than the minimal acceptable force.

The results of the FEM analysis showed that the maximal supraspinatus force was 11.6% higher than the minimal acceptable force. As such, the load would exceed twice the amount of maximal force required to tear the supraspinatus tendon, before the screw or the plate would show first signs of plastic deformation.

**Conclusion:**

Based on the results of this analysis and the fulfilment of our acceptance criterion, the FEM model indicated that the strength of the Kaisidis plate exceeded that of the proposed maximum loads under non-cycli loading conditions.

## 1 INTRODUCTION

Isolated fractures of the Greater Tuberosity (GT) of the humerus are among the most frequent shoulder injuries, and account for approximately 20% of all humeral fractures; 15-30% of all anterior glenohumeral dislocations result in a GT fracture [1, 2].

Although the magnitude of displacement which requires surgery remains debatable, most authors agree that fractures with more than 5 mm displacement in the general population, or more than 3 mm displacement in athletes and patients with frequent overhead activity, should be treated operatively [3-5].

The greater tuberosity is the insertion site of the supraspinatus, infraspinatus and teres minor tendons. Typically, a displaced greater tuberosity fracture is displaced proximally due to traction of the supraspinatus tendon, and posteriorly due to traction of the infraspinatus and teres minor tendons. Significant posterosuperior translation can cause malunion and impingement. Moreover, displacement of the rotator cuff insertion also modifies the force couple between the cuff tendons and deltoid muscle during shoulder abduction [6].

The optimal surgical fixation of greater tuberosity fractures remains unclear. Depending on the size and comminution of the tuberosity fragment, fixation can be achieved through heavy non-absorbable sutures, suture anchors, tension bands, screws with a washer or plates [7, 8]. In accordance with the recent literature, locking plate fixation provides the strongest biomechanical fixation for split type greater tuberosity fractures [9].

The goal of this study was to demonstrate the stability of a novel plate (Kaisidis plate, Fa. Königsee) for fixation of greater tuberosity fractures, taking into account that the supraspinatus tendon is the most influential factor.

## 2 MATERIALS AND METHODS

**Fig. (1):**
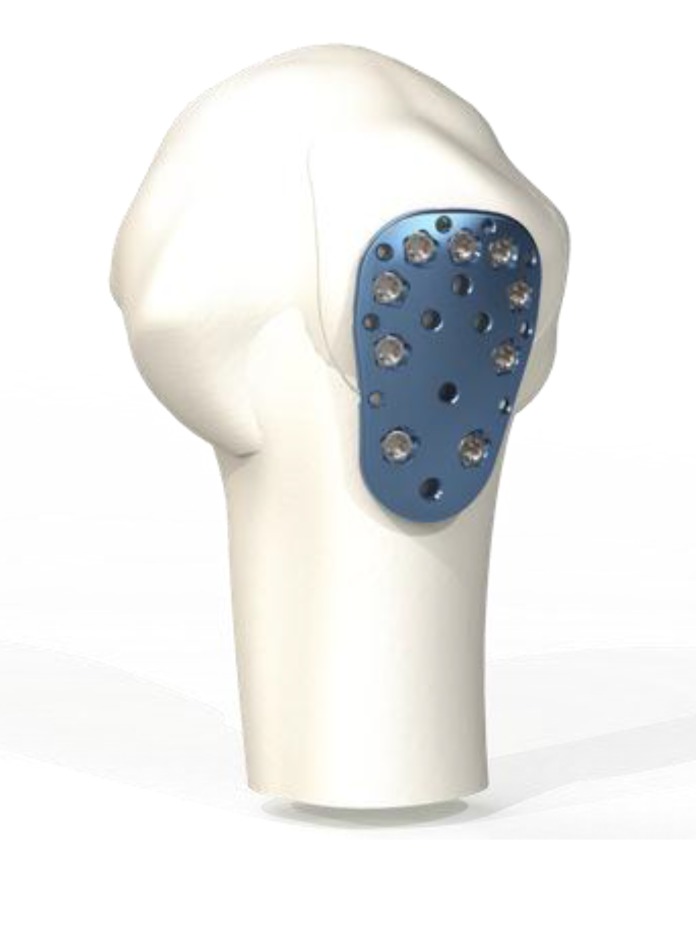
Kaisidis plate.

The goal of this analysis was to simulate the environment of application of the Kaisidis plate. The plate is judiciously applied up to the tuberosity edge, high enough to capture the tuberosity fragment (Fig. **1**). The Kaisidis plate is a bone plate intended for greater tuberosity fractures. It is a low profile plate with a length of 3.52 cm, width of 2.2 cm and thickness of 2.14 cm proximal and 2.45 cm distal. It has nine holes for 2,4 mm diameter locking screws, eight suture holes and additional K-wire holes for temporary fixation of the fragment. The Kaisidis Plate has multidirectional drill sleeves and no aiming device.

The scenario for such a fracture was created using SolidWorks 2015 software. SolidWorks is a solid modeling computer-aided design and computer-aided engineering computer program that runs on Microsoft Windows . SolidWorks is a solid modeler and utilizes a parametric feature-based approach to create models and assemblies [10].

**Fig. (2):**
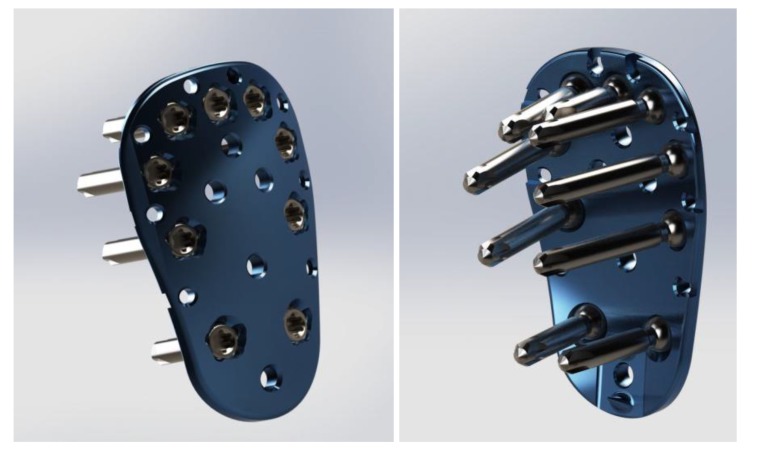
Kaisidis Finite Element Method model.

**Fig. (3):**
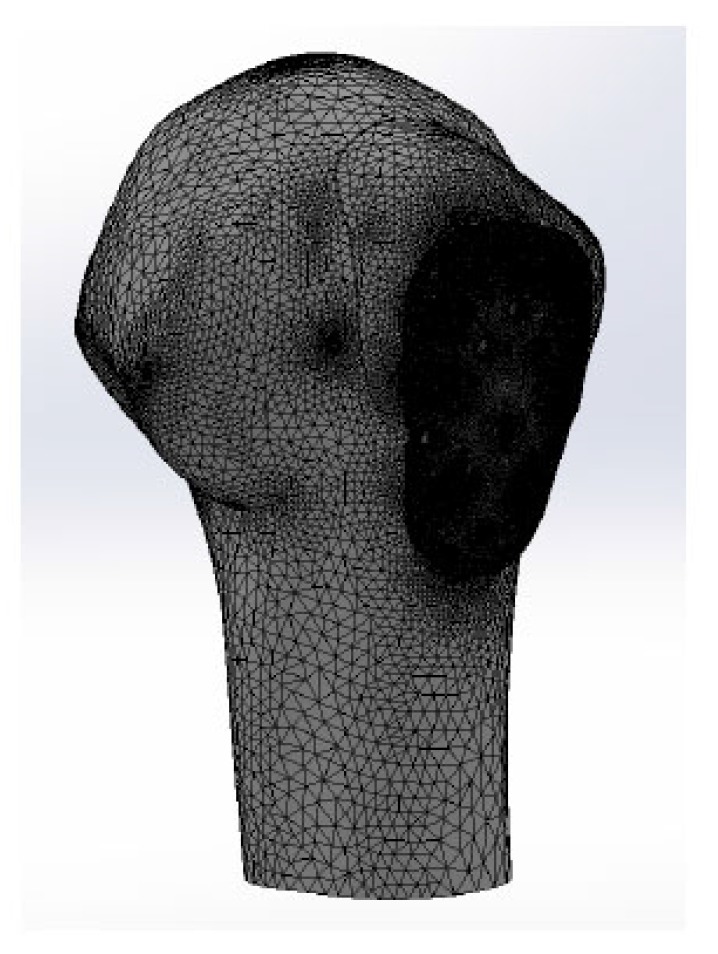
Finite Element Method setup meshing.

A Finite Element Method (FEM) simulation analysis was performed on a Kaisidis plate fixed with nine screws, in a greater tuberosity fracture model (Figs. **2** and **3**). The Kaisidis plate is made of grade 4 pure titanium. Self-tapping locking bone screws are made of titanium alloy. For this study, a simplified model of the Kaisidis plate and screws (T41431XX) with suppressed threads was used.

Three muscles were represented by loads, the supraspinatus muscle (Fss), subscapularis muscle (Fsc) and infraspinatus muscle (Fis). The test setup is shown in Fig. (**4**). The load on the fractured fragment is supplied by three muscles that are connected to the rotator cuff.

Of all the muscles involved, the supraspinatus is most involved in tearing. The subscapularis tendon with its insertion onto the lesser tuberosity has no effect on the stability of the fixation. Supraspinatus force confronts to the weight of the arm during abduction. The infraspinatus and teres minor generated forces play the role of stabilizing the upper limb during isometric abduction in the coronal plane [11]. Their directions of action do not resist the load of the arm during abduction. As a consequence, the supraspinatus generated force is taken in this simulation as the main force which has an impact on the greater tuberosity.

Additionally, infraspinatus and teres minor, both cover the back of the humeral head and are external rotators of the shoulder. In this simulation analysis, the influence of these two muscles is considered insignificant compared to the load produced by supraspinatus due to their insertion on the humeral head and their direction of action.

**Fig. (5):**
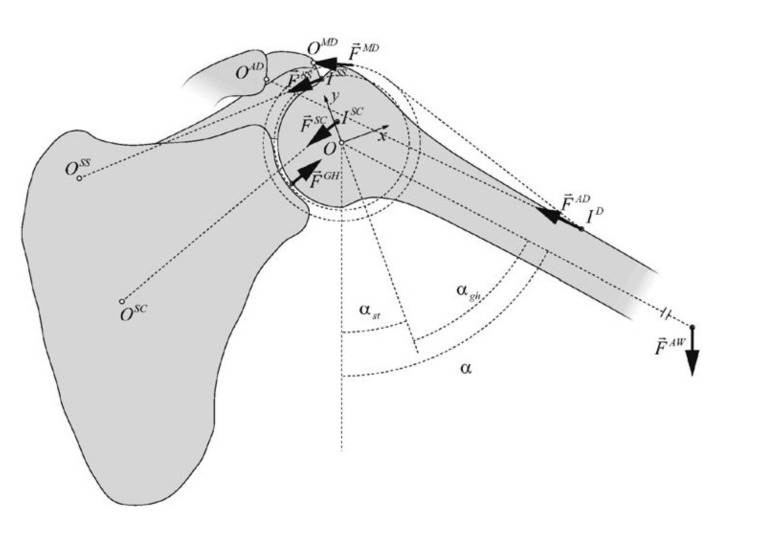
Force layout of rotator cuff.

**Fig. (6):**
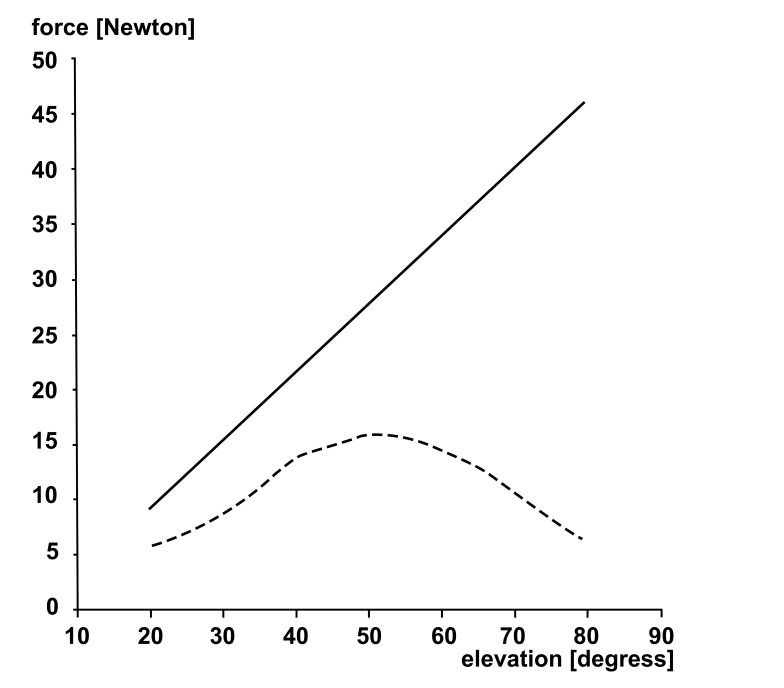
Distribution of supraspinatus force eliminated in various positions of glenohumeral joint elevation (solid line) and increase in deltoid force required to compensate for loss of supraspinatus force (dotted line).

For the purpose of this analysis, a setup is used with the assumption that the force of supraspinatus tendon is maximal and constant in every position of the arm. (angle a, shown in (Fig. **5**)). This situation is considered as impossible because the force distribution during abduction is not constant (Fig. **6**), but it is taken as a part of worst-case scenario.

Because the materials composing screws and plates have similar mechanical properties, and the difference in properties can be considered insignificant, one material was applied to both the screws and the bone plate. The model was meshed with parabolic tetrahedral solid elements. Table **2** presents a description of the model’s mesh. The maximal size of the elements was 10 mm, and the area where these elements were applied is the area where lowest stress occurs.

When a rotator cuff is used, three muscles supply load to the greater tuberosity. The supraspinatus tendon has the greatest effect on the fracture zone, and as such, it was the primary focus for this study. The maximal force value generated by supraspinatus (Fss) which is used in our simulation setup is the maximal possible force generated during an intense activity (Table **1**) [12]. In the cadaver, biomechanical testing performed by New Jersey Medical School, Newark, revealed that the peak recorded load where the supraspinatus tendons start to tear is 345 ± 6 N [13].

Based on this data, acceptance criteria were defined. As such, the maximum force generated from the supraspinatus Fss, at which the parts of the Kaisidis system show signs of yield, was designated to be two times higher than the maximal force recorded in biomechanical cadaver testing, that means that our system would not show yield until 690N. The force generated by the subscapularis and infraspinatus muscles was considered as a constant; for the subscapularis muscle, this value was 460 N, and for the infraspinatus muscle it was 200 N (Table **3**). Only linear calculations were performed.

## 3 RESULTS

The testing was performed in a way so that the total applied force would result in a maximum stress of 816 N/mm^2^. Our findings indicated that the most critical points of the Kaisidis system are the screws that are connected to the bone.

**Fig. (7):**
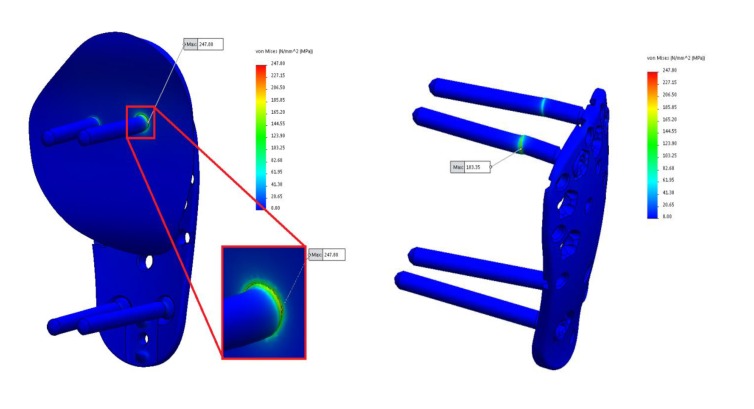
FEM results.

After the calculation has been done, applied force will result in a maximum stress of 183,35 N/mm^2^ located on the screw, and 247,80 N/mm^2^ located on the bone. From the (Fig. **7**) it can be seen that the most critical points of the Kaisidis system are screws.

**Fig. (8):**
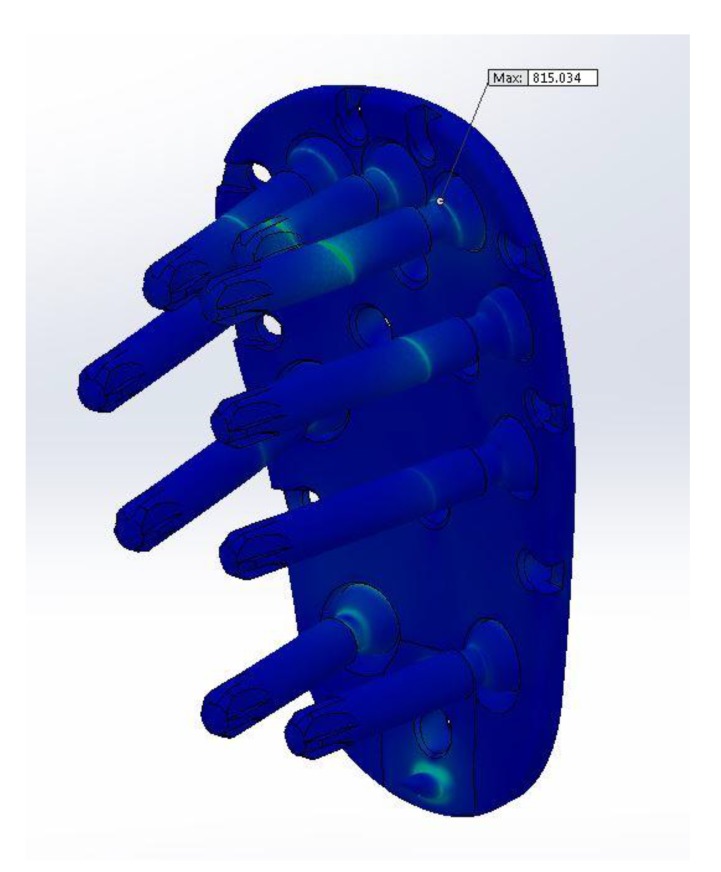
Finite Element Method results.

The maximal stress concentration is at the connection of the screw head and screw body (Fig. **8**). The maximal force generated by the supraspinatus was 784 N, which is higher than the minimum acceptable force. The minimally acceptable force by the supraspinatus was 702 N. It can be concluded that the maximal force that the supraspinatus needs to generate before the Kaisidis system is susceptible to plastic deformation is 11.6% higher than the minimal expected (Table **4**). Maximal generated stress by the system of forces which are acting on greater tuberosity is shown in Fig. (**9**). If yield strength of the material is compared to maximal detected stress, it can be concluded that safety factor for given load is S=4,25 (Table **5**).

From these results, it can be inferred that the load would exceed twice the amount of maximal force required to tear the supraspinatus tendon. Moreover, it can be seen, that the maximal generated force is acceptable in comparison with material properties and the Kaisidis system would withstand 4,25 greater load than presented in this analysis as the worst case scenario.

FEM analysis has also shown that the shear is the most dominant stress mechanism in the application of Kaisidis system and that the highest stress concentration is located on the body of the screws, near fracture line.

## 4 DISCUSSION

In the current biomechanical study, FEM simulation analysis showed that the Kaisidis plate fulfills the biomechanical requirements for the treatment of GT fractures, taking into account that the supraspinatus tendon was considered as the most influential stress factor. This analysis showed that this novel locking plate can provide anatomical reduction and rigid fixation of such a fracture. The fatigue strength of the plate was not considered in our simulation analysis because the forces in a recovering shoulder are quite low. The calculated stress was compared to mechanical properties of the material and it was concluded that the design and material properties of the Kaisidis system will withstand maximal possible load.

The quality of the cancellous bone used for fixation was not taken into consideration because these types of fractures occur in younger patients with good bone quality.

In the literature, it remains debatable as to which is the best treatment option for these fractures. The many options for treating these difficult injuries include suture fixation, percutaneous techniques, screw fixation, and more recently, arthroscopic suture techniques [14, 15]. The goal of any of these operative interventions is to restore normal function and minimize pain around the injured shoulder. Although most of the operative techniques for greater tuberosity fractures have predictable results, there is no one established technique considered as the gold standard for the treatment of displaced greater tuberosity fractures.

In general, patients who sustain an isolated greater tuberosity fracture are younger and more active than those who sustain other proximal humerus fractures [16]. As a result, in the treatment of GT fractures, there is increased emphasis on return to high activity and function. In three- and four-part fractures of the proximal humerus, plate osteosynthesis provides proximal suture fixation points and locking screws that can prevent suture cut-out and isolated screw failure. In addition, compared with other techniques for greater tuberosity fixation, meta-diaphyseal cortical plate fixation bypasses the often poor bone quality of the greater tuberosity, preventing these modes of failure. In the case of a large GT fracture, frequently the fracture line extends further distal of the surgical neck, making it difficult to reduce and fix the fracture arthroscopically with suture anchors.

Park *et.al*. reported on a novel arthroscopic-assisted anatomical plate fixation technique which was found to be effective in the treatment of comminuted or large-sized GT fractures, enabled an accurate restoration of the medial footprint of the fracture, and provided an effective buttress to the large fragments [17]. On the other hand, Lin *et.al*. reported that suture anchor constructs could be stronger than a rigid fixation through two screws [18]. However, it remains debatable if such a construct can effectively fix large and comminuted fragments, or reduce a posterosuperior displacement.

Schoffl and colleagues [19] reported on 10 patients who received a Bamberg plate; all 10 had excellent postoperative outcomes with no complications or secondary loss of reduction. In addition, Bogdan *et.al* [20]. found that using a mesh plate that can be contoured to the patient’s anatomy is a reliable alternative treatment method for the operative management of isolated greater tuberosity fractures.

Various operative treatment techniques for isolated greater tuberosity fractures have been described. Flatow and colleagues [21] reported an excellent return of forward elevation after offen open reduction and internal fixation with heavy suture, but only half of the patients reported excellent outcomes. Braunstein *et.al* [22]. examined the biomechanical strength of various fixation constructs and found that tension band wiring or cancellous screws were superior to suture fixation. With advances in arthroscopic instrumentation and techniques, displaced GT fractures may be treated with a suture anchor construct. Ji and colleagues [23] described encouraging outcomes after arthroscopic fixation of isolated displaced proximal humerus fractures in 16 patients.

However, there is another important issue concerning the treatment of GT fractures. The majority of these fractures are associated with partial or full thickness rotator cuff tears [24]. Thus, GT fracture fixation maintains rotator cuff tension and prevents further posterosuperior migration of the fragment [25]. The Kaisidis plate is a low profile, short length plate specifically designed for the reduction of isolated GT fractures. The plate allows sutures to be tied through it, in order to increase fixation strength and to distribute the strength of retention over a larger area.

It is difficult to secure anatomical reduction and rigid fixation for large, displaced GT fractures using tension band fixation, suture anchors or cannulated screws, because there is the risk of further fragmentation and displacement of the comminuted fracture fragments around the fixation device. The size of the fragment, the amount of comminution, and the displacement of the fracture are the most critical factors to consider in the treatment of these fractures.

## CONCLUSION

Based on the results of our analysis and the fulfilment of the acceptance criterion, it is evident that the Kaisidis plate possesses the required biomechanical stability. The maximal supraspinatus force was 11.6% higher than the minimal acceptable. From this result, it can be seen that the load would exceed twice the amount of maximal force required to tear supraspinatus tendons before the screw or the plate show first signs of plastic deformation. The maximal generated force is lower in comparison with material properties and the Kaisidis system would withstand 4,25 greater load than presented in this analysis as the worst case scenario. Further prospective, randomized clinical studies are needed to validate and confirm the clinical effectiveness of this novel plate.

## Figures and Tables

**Fig. (4):**
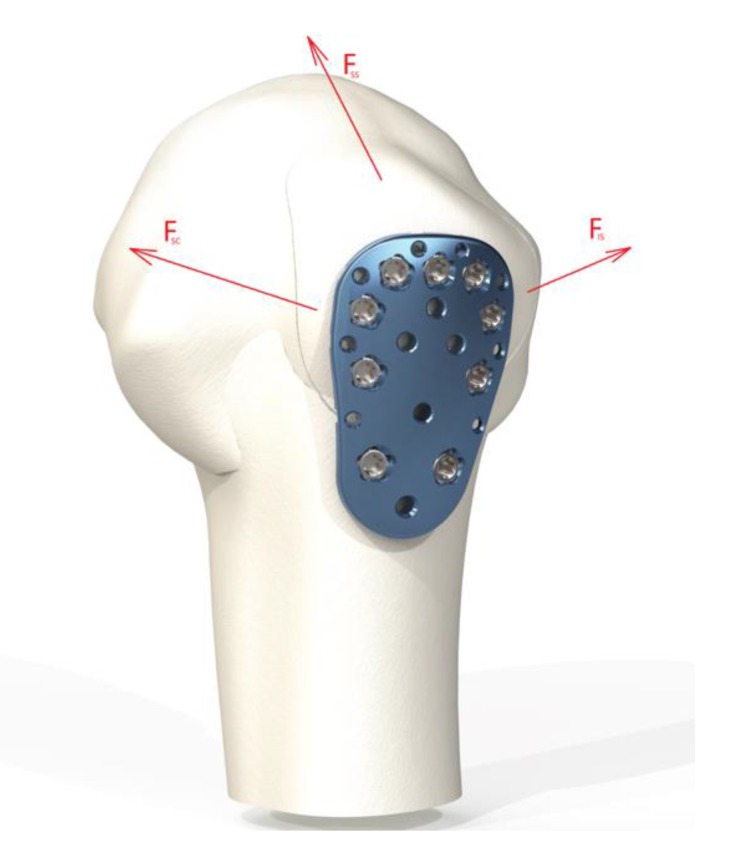
Finite Element Method test set up.

**Fig. (9):**
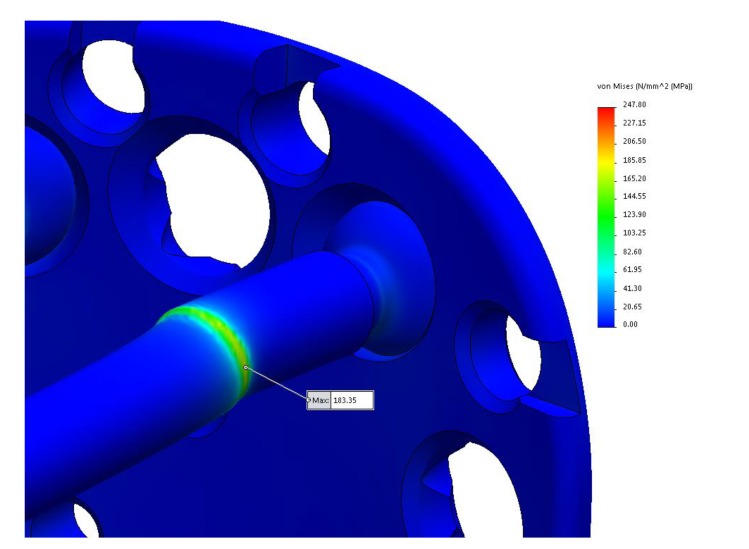
Maximal stress area.

**Table 1:** Forces transmitted through supraspinatus during various activities.

Activity	Force Transmitted Through Supraspinat**us**
During various exercises	156 N
During athletic activities	140-145 N
During maximum contraction of supraspinatus	196 N

**Table 2:** Mesh details.

**Number of Nodes**	**Number of Elements**	**Maximum Element Size**	**Minimum Element Size**
5251423	3782099	10 mm	0.1 mm

**Table 3:** Forces on the greater tuberosity.

**Muscle Force**	**Value in N**
Minimal acceptable force generated by the supraspinatus (F_SSmin_)	702
Force generated by the subscapularis (F_SC_)	460
Force generated by the infraspinatus (F_IS_)	200

**Table 4:** Test results.

**Minimal Acceptable Force (F_SSmin_)**	**Maximal Force (F_SSmax_)**	**F_SSmax/_ F_SSmin_**
702 N	784 N	1.116

**Table 5:** Test results.

Yield Strength of Material: Rp_0,2_	Maximal Detected Stress (von Mises): σ_max_	S=Rp_0,2 _/ σ_max_
780MPa	183,35 MPa	4,25
